# Establishment of a monoclonal antibody PMab-233 for immunohistochemical analysis against Tasmanian devil podoplanin

**DOI:** 10.1016/j.bbrep.2019.100631

**Published:** 2019-04-01

**Authors:** Yoshikazu Furusawa, Shinji Yamada, Shunsuke Itai, Takuro Nakamura, Junko Takei, Masato Sano, Hiroyuki Harada, Masato Fukui, Mika K. Kaneko, Yukinari Kato

**Affiliations:** a Department of Antibody Drug Development, Tohoku University Graduate School of Medicine, 2-1 Seiryo-machi, Aoba-ku, Sendai, Miyagi, 980-8575, Japan; b New Industry Creation Hatchery Center, Tohoku University, 2-1 Seiryo-machi, Aoba-ku, Sendai, Miyagi 980-8575, Japan; c ZENOAQ RESOURCE CO., LTD., 1-1 Tairanoue, Sasagawa, Asaka-machi, Koriyama, Fukushima, 963-0196, Japan; d Department of Oral and Maxillofacial Surgery, Graduate School of Medical and Dental Sciences, Tokyo Medical and Dental University, 1-5-45, Yushima, Bunkyo-ku, Tokyo, 113-8510, Japan

**Keywords:** Tasmanian devil podoplanin, PDPN, PMab-233

## Abstract

Monoclonal antibodies (mAbs) against not only human, mouse, and rat but also rabbit, dog, cat, bovine, pig, and horse podoplanins (PDPNs) have been established in our previous studies. PDPN is used as a lymphatic endothelial cell marker in pathological diagnoses. However, mAbs against Tasmanian devil PDPN (tasPDPN), which are useful for immunohistochemical analysis, remain to be developed. Herein, mice were immunized with tasPDPN-overexpressing Chinese hamster ovary (CHO)-K1 (CHO/tasPDPN) cells, and hybridomas producing mAbs against tasPDPN were screened using flow cytometry. One of the mAbs, PMab-233 (IgG_1_, kappa), specifically detected CHO/tasPDPN cells by flow cytometry and recognized tasPDPN protein by western blotting. Furthermore, PMab-233 strongly detected CHO/tasPDPN cells by immunohistochemistry. These findings suggest that PMab-233 may be useful as a lymphatic endothelial cell marker of the Tasmanian devil.

## Highlights

• PDPN is known as a specific lymphatic endothelial cell (LEC) marker.
• Sensitive and specific PMab-233 mAb against Tasmanian devil PDPN was produced.
• PMab-233 strongly reacted with Tasmanian devil PDPN in flow cytometry.
• PMab-233 is useful for IHC using paraffin-embedded cell sections.

## 1 Introduction

Podoplanin (PDPN), a type I transmembrane glycoprotein, is expressed in many cell types, including lymphatic endothelial cells [1]. Therefore, PDPN is extremely useful to distinguish lymphatic endothelial cells from vascular endothelial cells in pathological diagnoses [2]. We previously reported that C-type lectin-like receptor-2 (CLEC-2) is an endogenous receptor of PDPN [3,4]. Importantly, the PDPN-CLEC-2 interaction has been shown to facilitate the separation of embryonic blood and lymphatic vessels [5]. The expression of human PDPN (hPDPN) has been reported in several malignant tumors, including malignant brain tumors [[6], [7], [8], [9]], oral squamous cell carcinomas [10], pulmonary cancers [11], esophageal cancers [12], malignant mesotheliomas [13,14], osteosarcomas [[15], [16], [17]], chondrosarcomas [16], and testicular tumors [18]. The expression of hPDPN is associated with cancer metastasis and malignant progression [4,6,19]. To date, we have developed monoclonal antibodies (mAbs) against not only human [20] but also mouse [20], rat [21], rabbit [22], bovine [23], dog [24], cat [25], pig [26], and horse [27] PDPNs. Furthermore, an anti-cat PDPN mAb (PMab-52) cross-reacted with a tiger PDPN [28], and an anti-bovine PDPN mAb (PMab-44) cross-reacted with goat [29], sheep [30], and alpaca [31] PDPNs. However, *anti*-Tasmanian devil PDPN (tasPDPN) mAb has not yet been reported. In this study, we immunized mice with CHO/tasPDPN cells and established hybridomas that could produce mAbs against tasPDPN.

## 2 Materials and methods

### 2.1 Cell lines and animals

CHO-K1 and P3X63Ag8U.1 (P3U1) cells were obtained from the American Type Culture Collection (Manassas, VA, USA). The synthesized DNA of tasPDPN (accession No. XM_012545641.2) bearing an N-terminal PA16 tag (PA16-tasPDPN) was inserted into a pCAG-Ble vector (FUJIFILM Wako Pure Chemical Corporation, Osaka, Japan) [32]. The PA16 tag comprises 16 amino acids (GLEGGVAMPGAEDDVV) [33]. The CHO-K1 cells were transfected with pCAG-Ble vector containing PA16-tasPDPN using the Lipofectamine^®^ LTX and Plus™ reagent (Thermo Fisher Scientific Inc., Waltham, MA, USA). Stable transfectants were selected by limiting dilution and cultivated in a medium containing 0.5 mg/mL of Zeocin (InvivoGen, San Diego, CA, USA).

The P3U1, CHO-K1, CHO/tasPDPN, CHO/hPDPN [34], CHO/mouse PDPN (mPDPN) [34], CHO/rat PDPN (rPDPN) [21], CHO/rabbit PDPN (rabPDPN) [22], CHO/dog PDPN (dPDPN) [24], CHO/bovine PDPN (bovPDPN) [23], CHO/cat PDPN (cPDPN) [25], CHO/pig PDPN (pPDPN) [26], CHO/horse PDPN (horPDPN) [32], CHO/tiger PDPN (tigPDPN) [28], CHO/alpaca PDPN (aPDPN) [31], CHO/bear PDPN (bPDPN) [26], CHO/goat PDPN (gPDPN) [29], CHO/sheep PDPN (sPDPN) [30], and CHO/whale PDPN (wPDPN) [26] were cultured in a Roswell Park Memorial Institute (RPMI) 1640 medium (Nacalai Tesque, Inc., Kyoto, Japan), which was supplemented with 10% of heat-inactivated fetal bovine serum (Thermo Fisher Scientific Inc.), 100 units/mL of penicillin, 100 μg/mL of streptomycin, and 25 μg/mL of amphotericin B (Nacalai Tesque, Inc.). The cells were grown in an incubator at 37 °C with humidity and 5% CO_2_ and 95% air atmosphere. Female BALB/c mice (6 weeks of age) were purchased from CLEA Japan (Tokyo, Japan). The animals were housed under specific pathogen-free conditions. The Animal Care and Use Committee of Tohoku University approved all animal experiments.

### 2.2 Hybridoma production

We employed a Cell-Based Immunization and Screening (CBIS) method [25,33,35,36] to develop sensitive and specific mAbs against tasPDPN. Briefly, two BALB/c mice were immunized with CHO/tasPDPN cells (1 × 10^8^) intraperitoneally (i.p.) together with the Imject Alum (Thermo Fisher Scientific Inc.). The procedure included three additional immunizations, followed by a final booster injection administered ip. 2 days prior to the harvest of spleen cells. Subsequently, these spleen cells were fused with P3U1 cells using PEG1500 (Roche Diagnostics, Indianapolis, IN, USA), and the hybridomas were grown in an RPMI medium supplemented with hypoxanthine, aminopterin, and thymidine for selection (Thermo Fisher Scientific Inc.). The culture supernatants were screened by flow cytometry.

### 2.3 Flow cytometry

The cells were harvested following a brief exposure to 0.25% trypsin and 1 mM ethylendiaminetetraacetic acid (EDTA; Nacalai Tesque, Inc.). The cells were washed with 0.1% bovine serum albumin (BSA) in phosphate-buffered saline (PBS) and treated with primary mAbs for 30 min at 4 °C. Thereafter, the cells were treated with Alexa Fluor 488-conjugated anti-mouse IgG (1:2000; Cell Signaling Technology, Inc., Danvers, MA, USA) or Oregon Green anti-rat IgG (1:2000; Thermo Fisher Scientific Inc.). Then, fluorescence data were collected using the SA3800 Cell Analyzers (Sony Corp., Tokyo, Japan).

### 2.4 Determination of binding affinity by flow cytometry

CHO/tasPDPN was suspended in 100 μL of serially diluted PMab-233. Then, Alexa Fluor 488-conjugated anti-mouse IgG (1:200; Cell Signaling Technology, Inc.) was added. Fluorescence data were collected using the EC800 Cell Analyzer (Sony Corp.). The dissociation constant (*K*_D_) was calculated by fitting the binding isotherms to built-in one-site binding models in the GraphPad PRISM 6 (GraphPad Software, Inc., La Jolla, CA, USA).

### 2.5 Western blotting

Cell lysates (10 μg) were boiled in a sodium dodecyl sulfate (SDS) sample buffer (Nacalai Tesque, Inc.). The proteins were electrophoresed on 5%–20% polyacrylamide gels (FUJIFILM Wako Pure Chemical Corporation) and subsequently transferred onto a polyvinylidene difluoride (PVDF) membrane (Merck KGaA, Darmstadt, Germany). After blocking with 4% skim milk (Nacalai Tesque, Inc.), each membrane was incubated with primary mAbs, including 1 μg/mL of PMab-233, 1 μg/mL of anti-PA16 tag (NZ-1), or 1 μg/mL of *anti*-*β*-actin (AC-15; Sigma-Aldrich Corp., St. Louis, MO, USA), and subsequently with peroxidase-conjugated anti-mouse IgG (1:1000; Agilent Technologies, Santa Clara, CA, USA) or anti-rat IgG (1:10000; Sigma-Aldrich Corp.). The developed bands were visualized with the ImmunoStar LD (FUJIFILM Wako Pure Chemical Corporation) using the Sayaca-Imager (DRC Co. Ltd., Tokyo, Japan).

### 2.6 Immunohistochemical analyses

Cell blocks were produced using iPGell (Genostaff Co., Ltd., Tokyo, Japan) and processed to make 4-μm thick paraffin-embedded cell sections that were directly autoclaved in a citrate buffer (pH 6.0; Nichirei Biosciences, Inc., Tokyo, Japan) for 20 min. These tissue sections were blocked using the SuperBlock T20 (PBS) Blocking Buffer (Thermo Fisher Scientific Inc.), incubated with PMab-233 (1 μg/mL) for 1 h at the room temperature, and then treated with the Envision + Kit (Agilent Technologies Inc.) for 30 min. Color was developed using 3,3′-diaminobenzidine tetrahydrochloride (Agilent Technologies Inc.) for 2 min, and counterstaining was performed using hematoxylin (FUJIFILM Wako Pure Chemical Corporation).

## 3 Results and discussion

Most cancers are somatic in origin, and only a few transmissible cancers have been documented [37]. Transmissible cancers have been reported only in natural cases, such as canine transmissible venereal tumor in dogs [38] or devil facial tumor disease in Tasmanian devils [39]. Tasmanian devils (*Sarcophilus harrisii*) are endangered owing to the emergence of two clonally transmissible cancers: devil facial tumor disease 1 (DFT1) and devil facial tumor disease 2 (DFT2). DFT1 and DFT2 are infectious diseases that spread via biting [40]. DFT1 was first discovered in northeastern Tasmania in 1996 and has since then spread to more than 80% of the area across the island, causing a significant decrease in the population [41]. DFT2 was discovered in 2014 and is currently restricted to a small region of southeastern Tasmania [42]. Although we had previously developed mAbs against human [20], mouse [20], rat [21], rabbit [22], bovine [23], dog [24], cat [25], pig [43], and horse [27] PDPNs, mAbs against tasPDPN has not yet been developed. The development of *anti*-tasPDPN mAbs will enable us to perform pathophysiological studies about the lymphatic metastasis or lymphangiogenesis.

**Fig. 1:**
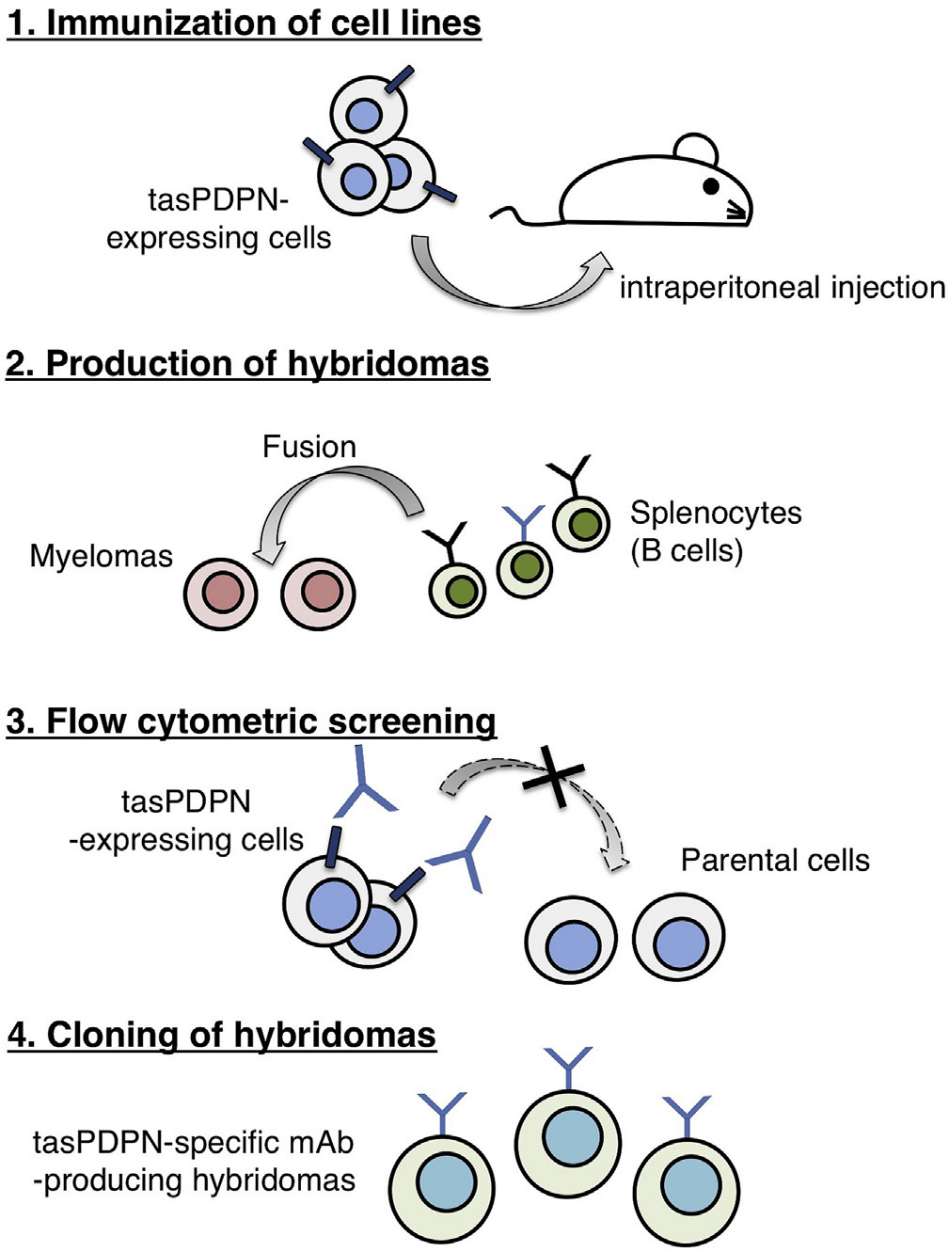
**Schematic illustration of the Cell-Based Immunization and Screening (CBIS) method.** Stable transfectants expressing the protein of interest are used as an immunogen with no purification procedure. The selection of hybridomas secreting specific mAbs is performed by flow cytometry using parental and transfectant cells.

In the present study, we employed the CBIS method to develop sensitive and specific mAbs against tasPDPN to facilitate the immunohistochemical analysis of paraffin-embedded tissue sections. Two mice were immunized with CHO/tasPDPN cells using an immunization and screening procedure (Fig. 1). The developed hybridomas were seeded into 96-well plates and cultivated for 9 days. Wells positive for CHO/tasPDPN and negative for CHO-K1 were selected by flow cytometry. The screening approach identified strong signals from CHO/tasPDPN cells and weak or no signals from CHO-K1 cells in 19 of the 960 wells (2.0%). After limiting dilution of 19 wells, we developed nine clones. One of these nine clones, PMab-233 (IgG_1_, kappa), was finally selected via immunohistochemistry against the paraffin-embedded sections of CHO/tasPDPN cell.

**Fig. 2:**
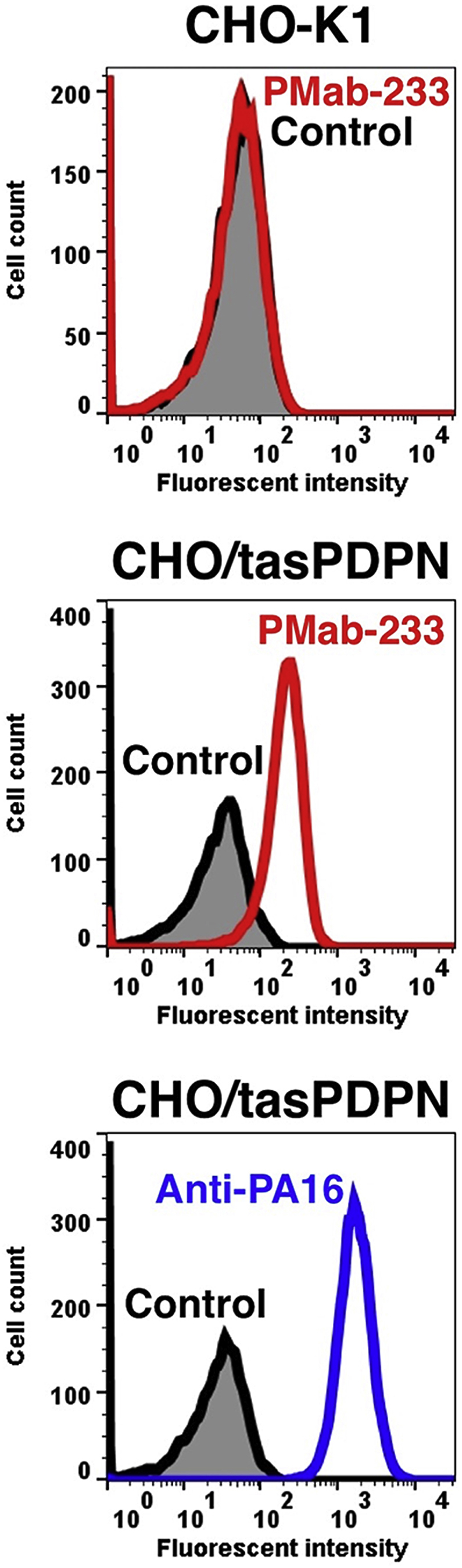
**Detection of tasPDPN by flow cytometry using PMab-233.** CHO/tasPDPN and CHO-K1cells were treated with PMab-233 (red line) or anti-PA16 tag (NZ-1; blue line) at a concentration of 1 μg/mL or 0.1% BSA in PBS (gray) for 30 min, followed by incubation with secondary antibodies.

**Fig. 3:**
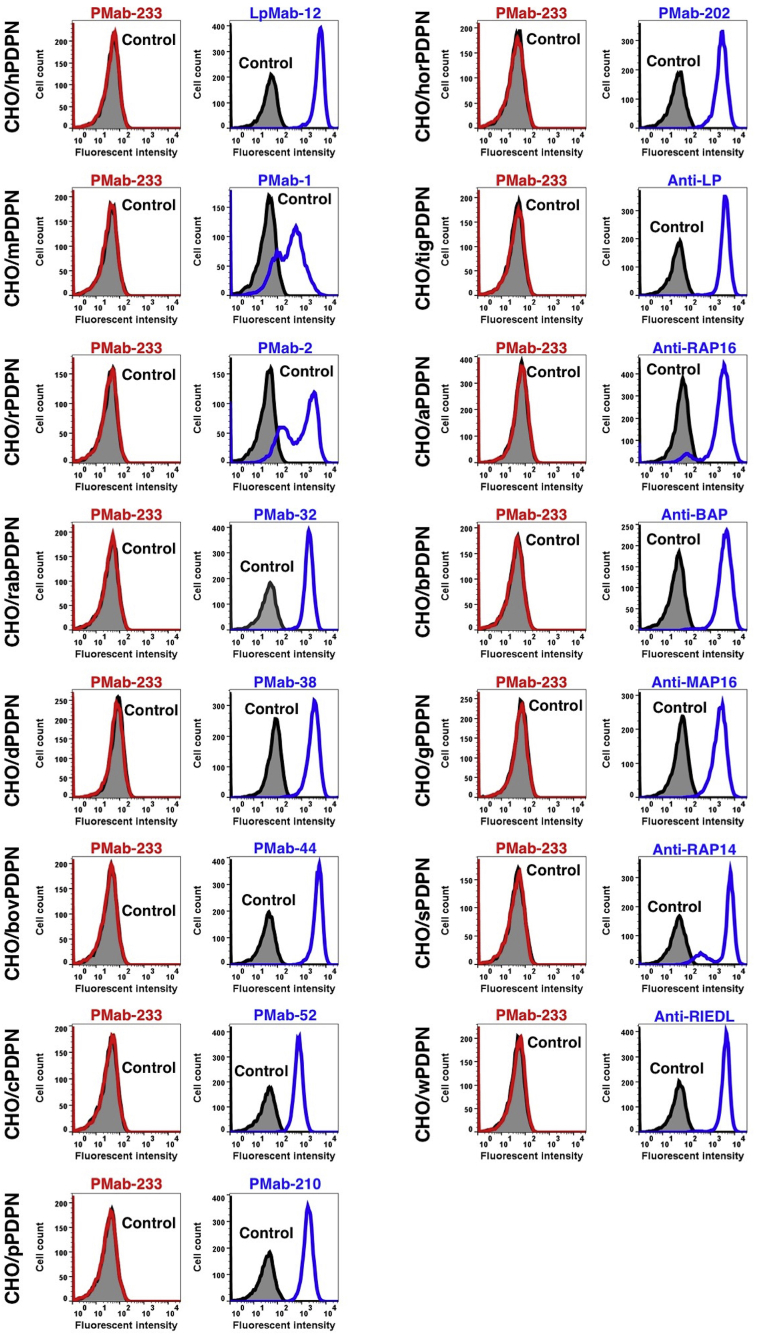
**Cross-reaction of PMab-233 to PDPNs of the other species by flow cytometry.** CHO-K1 cells transfected with PDPNs of the other species were treated with PMab-233 (red line) or each positive control (blue line) at a concentration of 1 μg/mL or 0.1% BSA in PBS (gray) for 30 min, followed by incubation with secondary antibodies.

PMab-233 recognized CHO/tasPDPN cells, but showed no reaction with CHO-K1 cells, as assessed by flow cytometry (Fig. 2). PMab-233 did not react with human, mouse, rat, rabbit, dog, bovine, cat, pig, horse, tiger, alpaca, bear, goat, sheep, or whale PDPNs (Fig. 3), which indicates that PMab-233 is specific to tasPDPN. The identity of PDPN amino acid sequence between tasPDPN and PDPNs of the other species is shown as below: 45% (vs. hPDPN), 41% (vs. mPDPN), 38% (vs. rPDPN), 36% (vs. rabPDPN), 45% (vs. dPDPN), 35% (vs. bovPDPN), 43% (vs. cPDPN), 39% (vs. pPDPN), 47% (vs. horPDPN), 44% (vs. tigPDPN), 49% (vs. aPDPN), 44% (vs. bPDPN), 39% (vs. gPDPN), 35% (vs. sPDPN), and 43% (vs. wPDPN).

In addition, kinetic analysis conducted by flow cytometry was employed to assess the interaction of PMab-233 with CHO/tasPDPN cells. *K*_D_ of PMab-233 for CHO/tasPDPN cells was determined to be 1.1 × 10^−6^, indicating a low affinity of PMab-233 for CHO/tasPDPN cells.

**Fig. 4:**
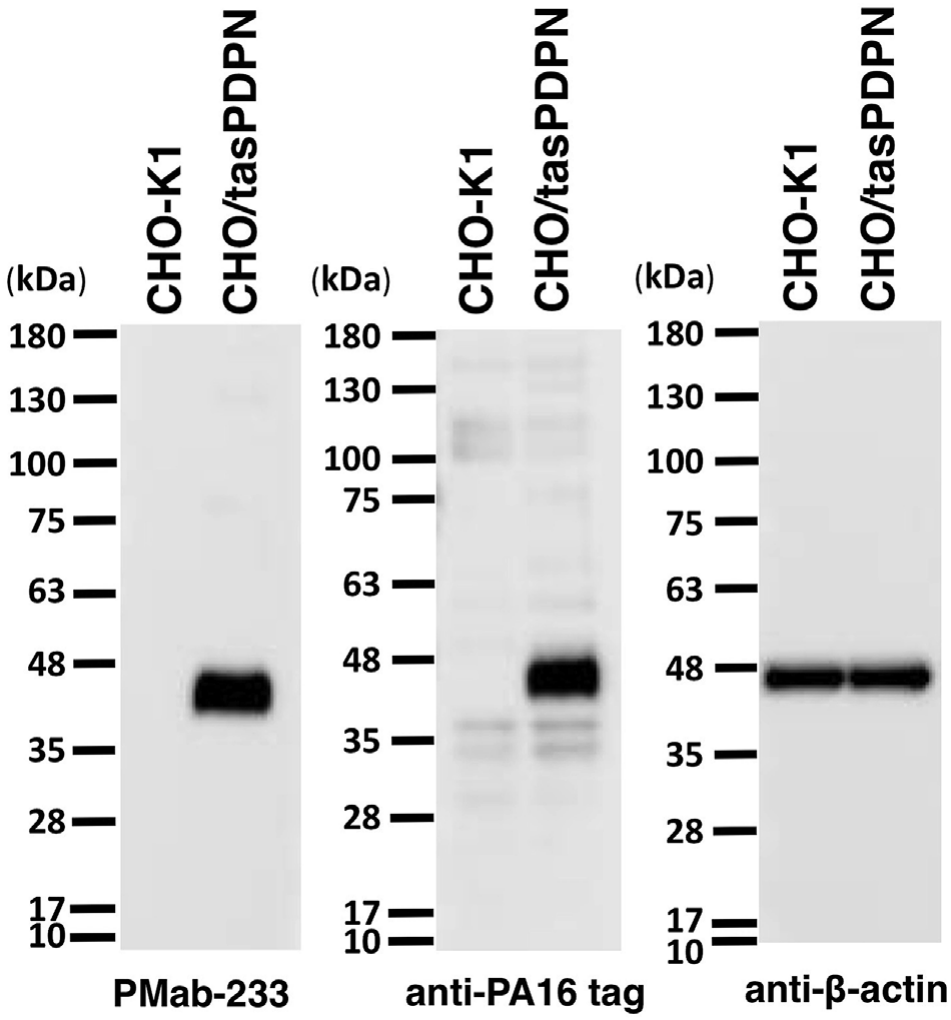
**Western blotting.** Cell lysates of CHO-K1 and CHO/tasPDPN (10 μg) were electrophoresed and transferred onto PVDF membranes. The membranes were incubated with l μg/mL of PMab-233, anti-PA16 tag (NZ-1), or *anti*-*β*-actin and subsequently, with peroxidase-conjugated anti-mouse or -rat IgG.

**Fig. 5:**
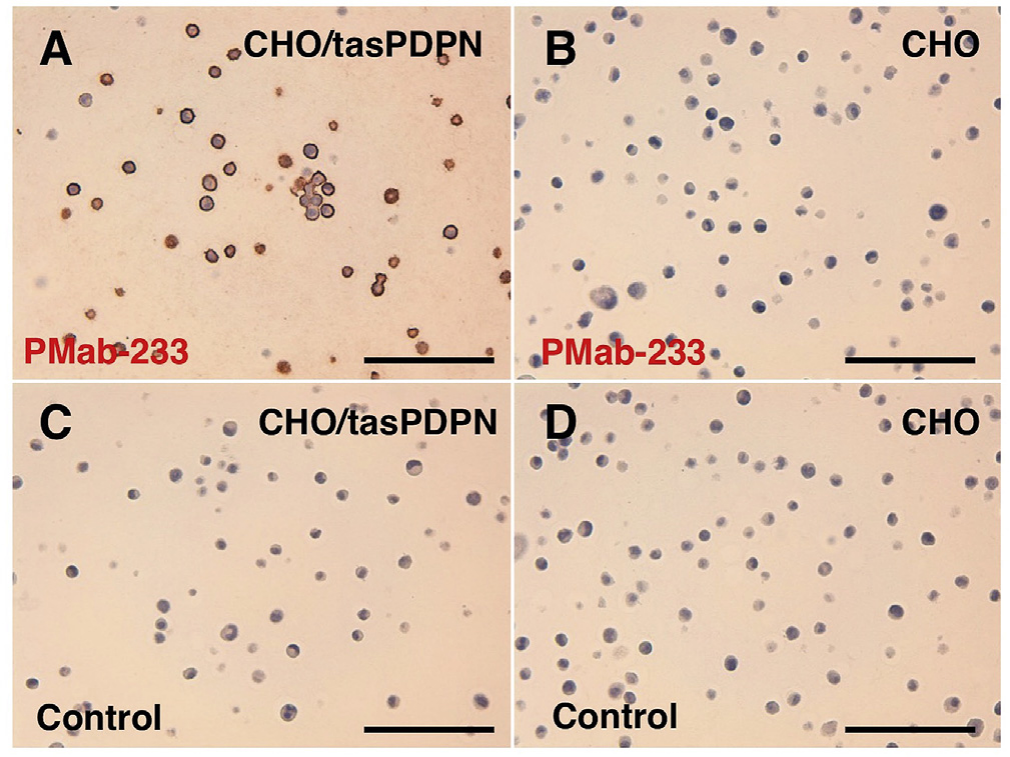
**Immunohistochemical analyses.** Cell sections of CHO/tasPDPN (A, C) and CHO-K1 (B, D) were incubated with 1 μg/mL of PMab-233 (A, B) or with blocking buffer (C, D), followed by that with the Envision + Kit. Scale bar = 100 μm.

Western blotting performed using PMab-233 (Fig. 4) demonstrated that PMab-233 detects tasPDPN as a 40-kDa band in CHO/tasPDPN cells. NZ-1, an anti-PA16 tag mAb also detected a 40 kDa band. The immunohistochemical analyses revealed that PMab-233 strongly stained CHO/tasPDPN cells (Fig. 5A) and did not react with CHO-K1 cells (Fig. 5B). No staining was observed without primary antibodies (Fig. 5C). These results cumulatively indicate that PMab-233 is useful for the detection of tasPDPN by immunohistochemistry.

In conclusion, we established an mAb, PMab-233, against tasPDPN, which is suitable for use in flow cytometry, Western blotting, and immunohistochemical analyses. The epitope of PMab-233 needs further investigation to clarify the sensitivity and specificity of PMab-233 against tasPDPN. We believe that PMab-233 should prove to be useful in elucidating the pathophysiological functions of tasPDPN in future studies.

## Conflicts of interest

Y.K. received research funding from ZENOAQ RESOURCE CO., LTD. The other authors have no conflict of interest.
